# The influence of clinic care on perceptions and knowledge of non-communicable diseases and physical activity from a low-resourced community: a mixed-method study

**DOI:** 10.1186/s12889-022-13097-w

**Published:** 2022-04-07

**Authors:** S. J. Makamu-Beteck, S. J. Moss, M. Cameron, F. G. Watson

**Affiliations:** 1 Physical Activity, Sport and Recreation, Faculty of Health Sciences, North-West University, Private Bag X6001, Potchefstroom, 2531 South Africa; 2 School of Health and Medical Sciences, University of Southern Queensland, Ipswich, 4305 Australia; 3 Qulaity in Nursing and Midwifery, Faculty of Health Sciences, North-West University, Potchefstroom, 2531 South Africa

**Keywords:** Non-communicable diseases, Physical activity, Standard clinic care health-promotion programme, Low-resourced African women perception, And knowledge, B-healthy

## Abstract

**Background:**

Health promotion for the management of risk factors for non-communicable diseases (NCDs) is an integral part of standard care in South Africa. Most persons presenting with NCDs utilise public primary health care centres for disease management. This mixed-methods study aimed at expanding current understanding of the the influence of standard clinic care (usual care) on perceptions and knowledge of risk factors for NCDs and physical activity (PA) among persons from a low-resourced community. Qualitatively the perceptions of women from a low-resourced community about risk factors for NCDs and PA were explored throughout 24-weeks of standard clinic care. Parallel quantitative data was collected to describe changes in risk factors for NCDs and trends in self-reported knowledge about risk factors of NCDs and PA.

**Method:**

A convergent-parallel mixed-methods research design was used. The study was carried out in a public primary health care setting, in the North West Province, South Africa. From a convenience sample of 100 participants, 77 African women aged between 34 and 79 years were recruited for the study. Data were collected at three time-points including baseline, 12 weeks, and 24 weeks of a standard clinic care health-promotion programme. The qualitative data was collected during focus group discussions, and the quantitative data included questionnaires on knowledge of physical activity and risk factors for NCDs as well as anthropometric and biological measurements. Qualitative and quantitative data were analysed independently for each phase and then consolidated for interpretation. All data was collected in the same setting.

**Results:**

Participants' initial understanding and perceptions of NCD risk factors were poor. Qualitative findings showed that participants knew little about the specific physical activity they could engage in and the role of PA in NCD management. Participants preferred low-intensity activities. Heart-disease knowledge improved significantly at 12 weeks intervention compared to baseline *MD* = -3.655, *p* < 0.001. There were improvements in PA knowledge at 12 weeks from baseline *MD* = -0.625 *p* = 0.02. There were significant weight (*MD* = 1.420, *p* = 0.002) and waist circumference reductions (*MD* = 0.621, *p* = 0.02) from baseline to 24 weeks.

**Conclusion:**

Standard clinic care improved knowledge of physical activity and risk factors for NCDs, but perceptions of risk factors for NCDs and PA were unchanged. This study offers insight into the perceptions held by women from a low-resource setting and how future interventions to manage and prevent NCDs should be structured.

**Trial registration:**

PACTR201609001771813.

## Background

In 2018, non-communicable diseases (NCDs) accounted for 59% of all deaths in South Africa [1]. Nearly two-thirds of South Africans, most of whom are treated for NCDs, depend on public health facilities (mainly primary care clinics) for out-patient health care [2–4]. The increased burden is managed through task shifting, which is the redirection of patient care from a specialist (e.g., cardiologist) to a nurse. Task shifting is initiated through the nurse-led clinics that are mid-level health providers [5]. In most cases, patients are seen by nurses, with support from doctors but rarely supporting staff such as dieticians, physiotherapists, or podiatrists [6]. Sixty-seven per cent of patients with NCDs were seen by nurses, while 33% were seen by doctors in four of the nine provinces of SA [4].

In South Africa, nurses tasked with health promotion are expected to implement behaviour-change strategies at clinics to manage the risk factors of NCDs. These programmes include educational posters, pamphlets, booklets, support groups, individual and group counselling [7]. An evaluation of a community-level NCD intervention revealed that employed persons who have pre-existing conditions are excluded from the interventions, because support groups were offered during working hours [8]. Our previous study that evaluated the point prevalence of NCD risk factors, the knowledge of NCDs and physical activity (PA), and perception about NCDs and PA among South Africans from rural, deep rural, and urban–rural areas in the North West and Northern Cape provinces of South Africa found a high prevalence of NCD risk factors coupled with low disease-specific knowledge and misperceptions about PA and NCDs. Because the previous study was an observational study, where health promotion was not implemented in the included public health clinics, exploratory research is needed to understand the effect of standard health-promotion clinic care on perceptions and knowledge of NCD risk factors and PA.

Perceptions and knowledge about non-communicable diseases influence people's behaviour toward health. The causes of NCDs are multifactorial, including an array of unhealthy lifestyle behaviours such as physical inactivity, tobacco use, alcohol abuse, and high-fat, low-fibre, high-sugar diets [5, 9]. Gaining a deeper understanding of the knowledge and perceptions of risk factors for NCDs among people dependent on the public health sector may assist with improving NCD management in these settings. Despite the importance of this topic, the literature is scarce on knowledge and perceptions of risk factors for NCDs and PA within the public health care sector.

The health belief model (HBM) predicts individual uptake of PA to avoid NCDs [10]. A scoping review by Sulat and colleagues asserted that the HBM variables are related to health behaviour [11]. The HBM includes six constructs, namely, (1) perceived susceptibility, (2) perceived severity, (3) perceived benefits, (4) perceived barriers, (5) cues to action and (6) self-efficacy. People will change their behaviour when they understand the risk of the disease and know about possible solutions to mitigate the risk. A few South African-based studies have effectively used the HBM to evaluate perceptions of NCDs [12–14]. The health belief model assisted to explain the variations in knowledge and behaviour and has the potential to describe perceptions and knowledge of NCDs and PA among participants receiving health promotion..

Patients' perceptions of health communication strategies delivered in primary health care centres have been described in the Free State province of South Africa [15]. Nyoni and Reid used semi-structured interviews to describe diabetic-related health communication strategies among patients in the Free State province of South Africa. They found that patients received information mainly on nutrition and lifestyle-modification strategies. However, the influence of primary health clinic standard care on patients' perceptions and knowledge of NCDs and its risk factors is not known, neither do we understand the effect of NCD support groups to inform on risk factors associated with NCDs, from the patient’s perspective. With this study, we aimed to explore, describe, and understand the influence of standard clinic care on perceptions and knowledge of risk factors for NCDs and PA among persons from a low-resourced community through a mixed-methods approach. Management of NCDs are influenced by knowledge and perceptions, as well as the presence of disease. Understanding the influence of standard clinic care for NCDs, will provide information on how to develop interventions for persons with NCDs living in low-resourced settings. 

## Methods

**Fig. 1:**
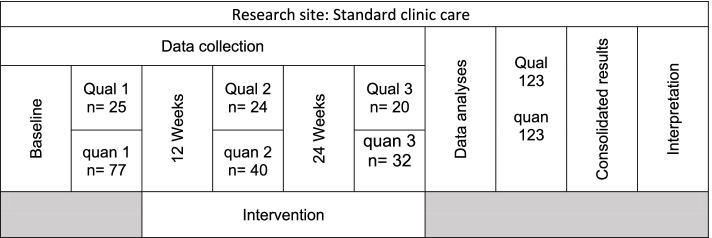
The mixed-methods approach of the study with qualitative (Qual) and quantitative (quan) data collection at three time-points and then consolidation and interpretation of the data

A convergent-parallel mixed-method design was followed (Fig. 1). Qualitative data and quantitative data were collected in parallel, analysed separately, and then consolidated for interpretation [16]. This study forms part of the B-Healthy controlled trial (PACTR201609001771813; Date of registration 7 September 2016) that introduced a supervised physical-activity intervention to persons dependent on a public health clinic for NCD support – and was compared to a standard clinic care health-promotion programme in a reference clinic support group. The setting was in Ikageng, a low-resourced community within the JB Marks Municipality of the Dr Kenneth Kaunda District in North West Province of South Africa. Ikageng comprises 98% Africans, whose first language is mainly Tswana, and to a lesser extent Sotho, Xhosa, and English. Lesego Clinic is a public primary health clinic offering standard NCD care in the form of NCD support groups to out-patients in Ikageng. The setting was selected, as the clinic provided the health promotion according to the nurse-led approach described as “task-shifting” in the primary health care setting of South Africa.

### Sampling and participants

Participants were recruited via convenience sampling from the catchment area of the Lesego Clinic. Ninety-one participants gave consent for the study; 14 participants were excluded for not meeting the inclusion criteria. Participants were excluded if they were either male, pregnant or lactating, had absolute contra-indications for exercise testing had orthopaedic limitation, had mental-developmental issues, or have access and use private health care. Participants were included if they were female with stable clinical condition, presenting with at least one cardiovascular risk factor, presented with low-to-moderate risk for participation in PA based on the physical activity readiness questionnaire (PAR-Q) [17].

### Standard clinic care

The standard clinic care health promotion programme aims to improve the health outcomes of persons diagnosed with an NCD or NCD risk factor [9]. Participants were asked to attend the clinic sessions scheduled by the nurse responsible for health promotion activities,, to attend workshops, and to receive medication. During the clinic sessions, the vital signs of the participants were measured. Participants were encouraged to form part of the support group. The support group attended workshops on NCD management, and an exercise session once a week, presented by one of the support group members. Nurses, Biokineticists, dieticians and general practitioners presented workshops on topics related to risk factors for NCDs, including cardiovascular risk factors, hypertension, diabetes, cardiac diseases, metabolic syndrome, obesity, nutrition and healthy diets, and leading active and sedentary lifestyles.

### Data collection

**Fig. 2:**
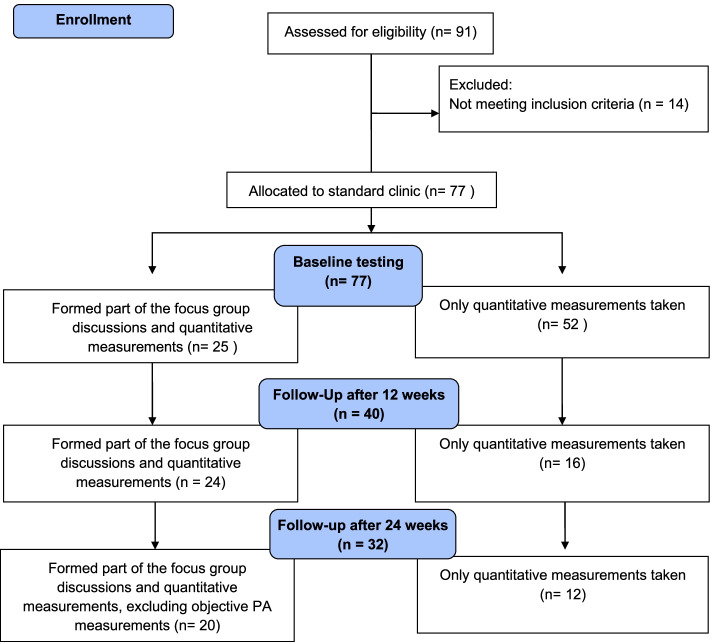
Schematic presentation of convergent mixed-method study

Qualitative and quantitative data were collected in parallel at baseline and after 12, and 24 weeks of standard clinic care. Qualitative explorations consisted of a minimum of four focus group discussions (FGDs) at each testing phase. A total of 13 FGDs consisting of four to ten participants per group were conducted with each discussion lasting no more than 60 min. The FGDs were scheduled with participants in a private room at the clinic where the support group met on a weekly basis.. Participants in the study were compensated for transport to the clinics.. From the total of 77 participants included at baseline, 25 participants contributed to FGDs at baseline. At 12 weeks of clinic care, 40 participants remained in the study – of which 24 contributed to the FGDs. During the 24 weeks of clinic care data collection, 32 participants remained in the study, of which 20 participants contributed to the FGDs (Fig. 2). The discussions were guided by an open-ended interview schedule that explored their perceptions and knowledge about risk factors for NCDs and PA. The concepts of 'individual perceptions', 'modifying factors', and 'likelihood of action' were addressed.

Discussions were conducted in English and translated into Tswana, by a researcher proficient in Tswana. Participants were encouraged to express themselves in Tswana to ensure maximum participation. All FGDs were audio-recorded. Discussions continued until data saturation was reached.

Quantitative measurements were collected to determine risk factors for NCDs, objective habitual physical activity, and knowledge of risk for NCDs and physical activity.. Body-composition variables included body mass and height measurements (seca scale, Hamburg, Germany) fro which body-mass index were calculated (kg/m^2^). Waist and hip circumference were measured with a steel tape (Cescorf, Porto Alegre, Brazil). Biological measurements collected were resting blood pressure, peripheral blood glucose, and peripheral cholesterol levels. Habitual PA levels were measured objectively for seven consecutive days employing combined heart rate and an accelerometry at baseline and 12 weeks (ActiHeart®, CamNtech Ltd, Cambridge, UK) [18]. Structural determinants consisted of heart disease knowledge [19], and PA knowledge adapted from Pienaar et al. [20] and previously used by [12]. The heart disease-knowledge questionnaire comprised 30 questions counting one point each, which could be answered by choosing true, false, or don't know. The percentage score attained for the questionnaires determining the knowledge was classified as low for 0–64%, moderate 65–69%, good for 70–79%, very good for 80–89%, and excellent 90–100%. A very good score suggests that a participant strongly grasps the subject matter, while a low score indicates an inability to grasp the subject matter. Cronbach's alpha coefficient for the PA knowledge questionnaire reliability was 0.66 and had 10 questions that are answered by encircling true, false, or unsure.

### Data analysis

The HBM was used as a lens for data analysis, which was performed separately at each time point. Qualitative data and quantitative data were mixed during the interpretation phase of the study. Critical points from qualitative and quantitative findings were consolidated, based on the constructs of the HBM.

Audio recordings of the FGDs were transcribed verbatim by an experienced transcriber proficient in both Tswana and English and analysed by the researcher with a computer-assisted qualitative data analysis software (CAQDAS) program (ATLAS.ti Scientific Software Development GmbH, Berlin, Germany) [21]. A non-linear process of noticing, coding and thinking (NCT) was followed to analyse qualitative data, based on predetermined themes: perceptions and knowledge of NCDs and perceptions, and knowledge of PA [22].

The Statistical Package for Social Sciences (SPSS) ver. 26.0 software (IBM SPSS Statistics, Chicago, IL, USA) was used for statistical analyses of the quantitative data. The distribution of data was determined using the Shapiro–Wilk test of normality. Descriptive analyses were performed, reporting medians (*Mdn*), interquartile range, means, standard deviations, and frequencies. Mauchly's test was conducted to test the assumptions of the repeated measures ANOVA and when the assumption of sphericity is violated, the Greenhouse-Geiser correction is applied. One-way repeated-measures ANOVA was used for normally distributed data while Friedman's test and Wilcoxon signed-rank test were performed when the assumptions of a repeated measures ANOVA were violated to determine any changes in PA knowledge, NCD risk-factor knowledge, habitual PA levels, and NCD risk-factor profile at baseline, 12 weeks, and 24 weeks of a standard clinic care programme. Post-hoc tests with Bonferroni adjustments were conducted to determine differing time points. The effect size reported when performing a repeated measures ANOVA for normally distributed data was determined using partial eta squared – where η_p_^2^ = 0.01 is small, η_p_^2^ = 0.06 is medium, and η_p_^2^ = 0.14 is large [23]. Non-normally distributed data effect size r was calculated from z statistic divided by the square root of the sample size [24]. Effect size was considered small if 0.1 ≤ *r* < 0.3, medium if 0.3 ≤ *r* < 0.5, and large if *r* ≥ 0.5 [23]. Findings were considered significant if *p* ≤ 0.05.

### Rigour

During the qualitative data collection, the researcher assumed a participatory role. Since the researcher is a health care professional applying PA as a modality for preventing and managing risk factors for NCDs, she engaged with the participants in their environment. The researcher also shares the same race as the participants and can speak their language. The researcher has training and experience in qualitative and quantitative research. Also, a more experienced researcher, the supervising researcher, analysed the transcripts while the student researcher analysed them independently, and the two sat and discussed the individual findings for consensus.

Methodological rigour in quantitative data collection was adhered to by ensuring that all researchers had training in the methods to be used. Standardised protocols for data collection were followed and measurements were taken twice for each participant. Double entry of data was done to ensure the correctness of capturing. The data set was checked for normal distribution skewness and kurtosis. Outliers were checked before data analyses.

## Results

The results from this exploration and description of perceptions and knowledge of NCD risk factors and PA, by participants from a low-resourced community exposed to standard health promotion clinic care for NCDs, are presented narratively.

A total of 77 women aged 34 to 79 years, mean age 61, SD ± 9.7 years, from a low resourced community, were included. The women were from impecunious households, with 83% reporting income less than R100 000 per annum. About two-thirds had secondary school education, while almost half were either unable to work due to a disability, illness or retired (Table 1). The constructs of the HBM were utilised to present the results for each measurement phase.

**Table 1:** Participants’ characteristics

**Variable**	**Value**
≥ 50 years (age)	86%
**Socio-economic variables**	
***Highest level of education:***	
No schooling	25%
High school	68%
Diploma	7%
***Employment status***	
Employed	21%
Unemployed	34%
Unable to work or retired	45%
***Marital status***	
Married	27%
Single	40%
Widowed	7%
Unspecified	26%
***Persons in household***	
1 – 3	43%
4 – 6	48%
> 6	9%
***Household income (annual)***	
Less than R100 000	83%
R100 000 – R250 000	9%
R250 000 – R400 000	8%

## Qualitative findings

### Baseline

#### Individual perceptions

The qualitative results revealed participants reported an increased risk of developing NCDs because of stress related to poor socio-economic factors. This finding is supported by the participants.

One participant stated, *“Stress causes NCDs”* (D1_01).

Participants mentioned that stress causes an array of physiological changes and ultimately leads to NCDs. One participant said, *“When you worry, high blood and sugar goes high and causes stroke”* (D3_02).

Another participant indicated *“*…*you find that there is no money, and you find that in the house we depend on one person, only one person getting pension”* (D4_02).

Participants attributed unhealthy dietary choices to lack of money.

One participant remarked, *“Unemployment makes us eat anything”* (D2 _01).

Participants also reported that dietary changes brought by modernisation increase NCD risk.

One participant remarked, *“We don't eat the leafy vegetables we used to eat”* (D1_01).

#### Modifying factors

The results further reveal that participants have limited knowledge about NCDs. Some participants reported that they knew of NCDs only after being diagnosed with an NCD condition.

One participant reported, *“I only knew about diabetes when the doctor told me I had diabetes”* (D1_01). Another participant explained, *“The doctors might not see diabetes, then one-day high blood rises, and diabetes shows”* (D2_01).

However, this lack of knowledge participants articulated may lead to some level of distrust for diagnoses by medical professionals. This maybe driven by cultural perceptions of disease symptoms of NCDs and the associated risk factors.

Participants expressed low self-efficacy for NCD prevention. They believed having a family member who is diagnosed with an NCD condition causes the entire family to have the disease, describing NCDs as family conditions.

A participant expressed disbelief of test results given by medical professionals: *“The doctors don't see diabetes in me though my grandmother and father had diabetes” (D3_02).*

However, medical professionals play a major role in motivating participants to initiate PA.

One participant recounted, *“When I went to the doctor, he sat me down and explained what fat does to the body and asked me to exercise to reduce fat”* (D3_02). She further remarked, *“People in my neighbourhood think we are stupid when they see us exercising, but I have reaped benefits. I lost weight”* (D3_02)*.*

This response indicates that the population may be subjected to discouragement from the local community regarding PA engagement.

#### Likelihood of action

The results indicate that participants experience many barriers to regular moderate-intensity PA engagement. Participants expressed a lack of social support to engage in PA from others. One disheartened participant mentioned, *“They say I am old; I am going to die, so it won't make a difference” (D3_02).*

Notwithstanding their concerns about not knowing which physical activities they could engage in, a participant questioned, *“What is physical activity?”* (D1_01).

Low energy levels hindered some participants from regular PA. A participant said, *“I have short breath, I can't walk fast, I lose my breath and sweat often” (D2_01).*

Even within a group setup low energy levels reduced opportunities for PA. A participant recalled, *“There was a young person that helped us to exercise. He got irritated, saying the elderly like to sit. If you feel you are tired, you sit” (D4_02).*

Participants reported awareness of the health benefits of regular PA. Participants preferred PA within a group, mainly for the social benefits, as reflected by one response: *“Gym is not only physical but also talking with others, relieves stress when we are together”* (D4_02). Participants indicated that they usually engage in activities within the traditional gender roles, such as house chores.

The preferred time of day for PA is generally early morning hours; a participant indicated, *“Waking up early makes you healthy” (D1_01).*

### Twelve weeks of standard clinic care

Results from the FGDs conducted at 12 weeks of standard clinic care, again interpreted against the HBM, indicated an increased perceived risk and perceived seriousness of NCDs.

#### Individual perceptions

The results indicate that participants perceived that everyone is at increased risk of developing NCDs because of modernisation.

One participant exclaimed, *“We are killed by things of nowadays”* (D10_01). Participants reflected on the past, reporting that the use of traditional medicine was protective against NCDs. One said, *“In olden days, NCDs were not there. Only flu and you were given serokolo (wild ginger – siphonochilus aethiopicus)”* (D10_01)*.*

Another said, *“We used to drink home remedies and be fine without going to the doctors”* (D10_01).

Participants perceive that change in diet fuel NCDs. They mentioned that the traditional diet was healthy and protected against NCDs. One participant said, *“People in the olden days used to eat leafy vegetables; these days we eat tinned stuff”* (D12_01).

Poor stress management was perceived as the cause of uncontrollable risk factors which cause NCDs. One participant emphasised, *“Always being hurt, staying with high emotions causes high blood and diabetes”* (D12_01). Another participant remarked, *“*I got diabetes because of worrying*”* (D10_01).

At 12 weeks, participants were able to perceive the seriousness of NCDs; they mentioned that NCDs can result in mortality. One participant explained, *“When your high blood is high in the brain, you get stroke”* (D11_01). Another participant reported, *“You can die from getting stroke, high blood, and diabetes”* (D11_01).

#### Modifying factors

Participants indicated that NCDs are new to them. One said, *“We are told we have high blood during the tests at the clinic”* (D10_01)*.*

They mentioned that their parents did not have an NCD condition, consequently they lacked prior NCD knowledge.

One participant said, *“We don't know anything about high blood and sugar because our parents didn't have those diseases”* (D10_01).

The results indicate that participants do not understand the role of PA in health, regardless of age. One participant reported, *“Exercise will bring down disease if you are young”* (D12_01). Participants perceived missed opportunity for PA engagement, as indicated by one response: *“We didn't exercise before”* (D12_01).

Mass media campaigns may be effective in raising awareness to adopt PA for NCD management. One participant reported encouragement through media. She said, *“After hearing from TV that you can level diabetes with exercise, I said I will gym with the support group”* (D12_01).

#### Likelihood of action

Participants reported benefits of PA engagement, including improved health and stress management. *“When we gym together the stress goes”* (D13_01).

Referring to health improvements, others said, *“If you exercise and take your treatment, high blood is controllable”* (D11_01). Another participant reported, *“I no longer go to the doctor as often for my knees since I started exercising”* (D12_01).

Though participants do not perceive barriers to PA engagement, the results indicate a significant presence of cultural barriers to PA. Participants define PA within the context of walking and housekeeping, and seldom mention recreational activities. One participant described, *“Home chores like washing the dishes is part of exercise”* (D11_01). Another participant indicated, *“Walking is an important part of exercise”* (D12_01). These responses suggest that participants mainly engage in low-intensity activities.

### Twenty four weeks of standard clinic care

The same procedure was followed at 24 weeks for data collection. The results showed that participants perceived that the elderly are at increased risk. However, most participants perceived NCDs as not life-threatening.

#### Individual perceptions

Participants perceived that older people are at increased risk because they are less active, compared to young people. One participant said*, “Elderly people are prone to NCDs since they are not able to do what they used to do when they were younger”* (D7_01).

They mentioned that NCDs are conditions associated with affluence, referring to NCDs as *“*Tycoons’ sicknesses*”*, which are now prevalent among all people mainly because of stress and unhealthy diets. One participant said, *“NCDs are tycoons’ sicknesses (wealthy people) since they could afford food that we were not able to buy”* (D7_01).

Participants reported that stress causes NCDs, a participant mentioned, *“I was always getting angry and shouting, I ended up getting diabetes”* (D5_01).

The findings revealed that participants perceived that NCDs are illnesses that affect an individual over a long period and may require specialised care in a hospital. One participant said, *“My high blood and sugar was high, I collapsed and was admitted to the hospital for a week”* (D5_01). Another participant explained, *“NCDs make you sick but will not immediately kill you”* (D9_02).

#### Modifying factors

The results indicate that participants have a low internal locus of control which may be attributed to an insufficient understanding of NCD risk factors. One participant remarked, *“If we knew where it is coming from, we would try to prevent it”* (D9_02).

Participants have basic knowledge of NCDs. They were unable to comprehend the causes for changes in medical test results received at primary health centres. One participant elaborated, *“At times at the clinic, they say my sugar is level, but my high blood is high”* (D9_02). Besides being diagnosed with an NCD condition or risk factor, they also were aware of family members diagnosed with NCDs.

Participants were aware of the role of PA in stress management. One participant said, *“PA helped me reduce stress”* (D7_01). They were confused about the role of PA in disease management. One participant said, *“Exercise helps control diabetes”* (D6_01). Another participant remarked, *“For me, I don’t see PA helping for my condition”* (D5_01).

#### Likelihood of action

Participants were not aware of the type of physical activities in which they could engage. They physically demonstrated what they perceived as PA, *“We gym like this with our hands and all that”* (D7_01). Another participant said, *“Even if it’s hard, you will do this. Even if you go down all the way, you will just do it”* (D5_01).

Participants perceived the benefits of PA as mainly related to energy levels. One participant elaborated, *“I can walk fast for long distances without getting tired, I am an expert of pacing”* (D6_01).

Participants preferred to engage in PA in the morning. One explained, *“Stretching is what you should do when you wake up, don’t just wake up”* (D7_01).

PA activities included house chores. A participant explained, *“That is exercising, isn’t we work with our hands. As a woman working in the house doing everything”* (D9_02).

## Quantitative findings: modifying factors

### NCD risk factors prevalence

**Fig. 3:**
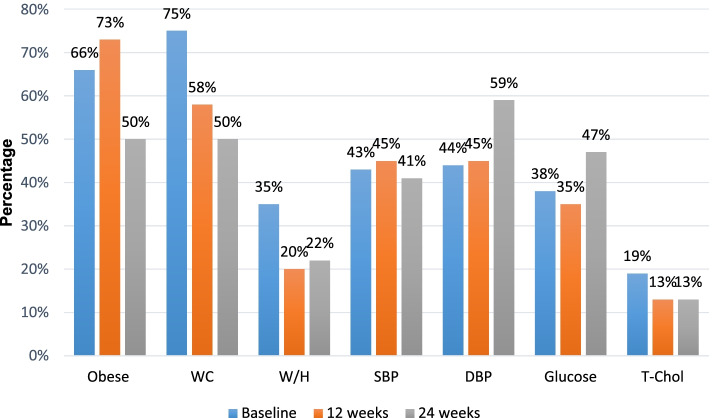
Percentage participants presenting with non-communicable disease risk factors at baseline, 12 and 24-weeks of standard clinic care

Many of the participants included in this study sample presented with NCD risk factors. The risk factors (Fig. 3) include obesity (BMI ≥ 30 kg/m^2^), waist to hip ratio (≥ 0.86), waist circumference (≥ 88 cm), systolic blood pressure (≥ 130 mmHg), diastolic BP (> 80 mmHg), glucose (≥ 5.6 mmol/L), and total cholesterol (≥ 5.2 mmol/L). Obesity was the most prevalent NCD risk factor, while total cholesterol was the least prevalent NCD risk factor among participants.

DBP = diastolic blood pressure, SBP = systolic blood pressure, T-Chol = total cholesterol; WC = waist circumference, W/H = waist to hip ratio.

Waist circumference was the most prevalent risk factor among participants at baseline accounting 75%, followed by obesity (66%). However, at 12 weeks, obesity was the highest prevalent risk factor (73%) and waist circumference reduced to 58% compared to baseline. At 24 weeks prevalence of obesity (50%) and waist circumference (50%) was the lowest compared to baseline and 12 weeks. The prevalence of waist-to-hip ratio remarkably reduced from baseline (35%) to 12 weeks (20%) and stabilised at 24 weeks (22%). Less than half of the participants had high SBP at baseline (43%), increased by two percent at 12 weeks (45%) and reduced by two percent from baseline to 24 weeks (41%). Regarding prevalence of high blood pressure, hypertensive DBP was below average at baseline (44%) and 12 weeks (45%) but above average at 24 weeks (59%). The prevalence of blood glucose decreased from baseline (38%) by three percent at 12 weeks (35%) but increased at 24 weeks (47%). The prevalence of total cholesterol was very low, accounting for 19% at baseline and reduced to 13% at 12 weeks and 24 weeks.

### Results of the changes in NCD risk factors from baseline, to 12 weeks and 24 weeks

Results from the repeated measures ANOVA showed a significant difference between the time points for weight, *F* (2, 56) = 10.125, *p* < 0.001, η_p_^2^ = 0.27 (Table 2). Post hoc pairwise analyses using the Bonferroni correction were significant between baseline and 24 weeks (*p* = 0.002), 12 to 24 weeks (*p* = 0.004) but not significant between baseline to 12 weeks (*p* = 1.000).

**Table 2:** Risk factors for non-communicable diseases at baseline, 12 and 24 weeks of a standard clinic care programme in a low-resourced community

**NCD risk factor**	**N**	Baseline (mean ± SD or median [IQR]*	12 weeks (mean ± SD or median [IQR]*	**24 weeks (mean ± SD or median [IQR]**^a^	***P***	**Effect size (partial n**^**2**^** or r)**^b^
Weight (kg)	29	79.3 ± 18.2	79.2 ± 18.2	77.9 ± 17.4	< 0.001	0.27
BMI (kg/m^2^)	29	32.7 ± 8.3	32.7 ± 8.3	32.7 ± 9.3	0.947	0.00
Waist (cm)—Transformed	29	94.6 ± 14.0	87.9 ± 13.0	87.7 ± 15.5	0.003	0.23
W/H	29	0.82[ 0.78 – 0.88]	0.79[ 0.75 m – 0.85]	0.81[0.77 – 0.85]	< 0.001	-0.30
SBP (mmHg)	29	123[116 – 139]	127[118—135]	134[118—156]	0.441	0.03
DBP (mmHg)	29	79 ± 14	77 ± 10	84 ± 12	0.005	0.17
Glucose (mmol/L)	29	4.8[3.9 – 5.7]	4.8[4.1 – 6.4]	5.6[5.0 – 6.8]	0.782	0.01
T-chol (mmol/L)	28	4.45 ± 0.98	4.14 ± 0.87	4.20 ± 0.71	0.040	0.11
PAL	26	1.39[1.320 – 1.520]	1.49[1.270 – 1.990]	-	0.053	0.16
Activity (counts/min)	26	19.70[15.30 – 26.60]	18.60 [13.40 – 25.50]	-	0.089	-0.14
AEE (kCal)	26	383.00[286.00 – 494.00]	502.00[234.00 – 1247.00]	-	0.038	0.17
MVPA (min/day)	26	25.00[11.00 – 42.00]	40.00[14.00 – 158.00]	-	0.030	0.18
Actiheart (worn days)	26	6[6 -6]	6[6 -6]	-	0.829	-0.02
Heart disease knowledge (score)	29	11 ± 4	15 ± 3	14 ± 3	< 0.001	0.29
Physical activity knowledge (score)	28	9[8-10]	10[9-10]	9[9-9]	0.007	0.24

*AEE* Activity energy expenditure, *BMI* Body-mass index, *DBP* Diastolic blood pressure, *MVPA* Moderate-to-vigorous physical activity, *PAL* Physical activity level, *SBP* Systolic blood pressure, *T-Chol* Total cholesterol, *W/H* Waits-to-Hip ratio

^a^Mean and standard deviations were used for variables that were normally distributed. The median and interquartile range was used for non-normally distributed variables

^b^partial n^2^ were used for repeated measures ANOVA, effect size r was used for non-parametric data

A Friedman’s test of waist-to-hip ratio was conducted. There was a statistically significant difference in baseline, 12 weeks, and 24 weeks x^2^(2) = 13.724, *p* < 0.001 (Table 2). Post hoc analysis with Wilcoxon signed-ranks tests showed that waist-to-hip ratio measurements at 12 weeks *Mdn* = 0.79 were lower than baseline scores *Mdn* = 0.82 resulting in a significance difference of z = -3.696, *p* < 0.001, *r* = -0.30.

In the case of waist circumference, Mauchly’s test indicated that the assumptions were not met x^2^(2) = 11.773, *p* < 0.003. Degrees of freedom were corrected using Greenhouse–Geisser. There was a significant effect, F (1.478, 41.377) = 8.171, *p* = 0.003, η_p_^2^ = 0.23 (Table 2). Post hoc tests using Bonferroni’s pairwise comparison show a non-significant difference between baseline and 12 weeks (1.000), but significant differences between baseline and 24 weeks (*p* = 0.015) and between 12 and 24 weeks (*p* = 0.013).

For diastolic blood pressure there was a significant change over time, F (2, 56) = 5.760, *p* = 0.005, η_p_^2^ = 0.171 (Table 2). Bonferroni's post hoc tests showed non-significant differences between baseline and 12 weeks (*p* = 1.000), and baseline and 24 weeks (*p* = 0.066). However, there was a significant difference between 12 and 24 weeks (*p* = 0.005).

In the case of total-cholesterol there was a significant effect, F (2, 54) = 3.420, *p* = 0.040, η_p_^2^ = 0.112 (Table 2). Post hoc tests using Bonferroni’s pairwise comparison show non-significant differences between all measurement time-points: baseline and 12 weeks (*p* = 0.090), baseline and 24 weeks (*p* = 0.226) and between 12 and 24 weeks (*p* = 1.000).

#### Habitual physical activity levels

The Wilcoxon signed-ranks tests on the habitual physical activity variables indicated significant differences between baseline and 12 weeks measurements of MVPA and AEE. MVPA levels were significantly higher at 12 weeks (Mdn = 40.00 min/day) than baseline (Mdn = 25.00 min/day), z = 2.172, *p* = 0.030, *r* = 0.18. Similarly, AEE levels were higher at 12 weeks (502.00 kCal) than at baseline (383.00 kCal), z = 2.070, *p* = 0.038, *r* = 0.17 (Table 2).

Results of the repeated measures ANOVA for heart disease knowledge showed a significant effect, *F* (2, 56) = 11.680, *p* < 0.001, η_p_^2^ = 0.294 Table 2. Post hoc pairwise analyses using Bonferroni were significant between baseline and 12 weeks (*p* < 0.001), baseline to 24 weeks (*p* = 0.010) but not significant between 12 to 24 weeks (*p* = 1.000).

A Friedman’s test of PA knowledge was conducted. There was a statistically significant difference in baseline, 12 weeks, and 24 weeks x^2^(2) = 9.787, *p* = 0.007 (Table 2). Post hoc analysis with Wilcoxon signed-ranks tests showed that PA knowledge scores at 12 weeks *Mdn* = 10 were higher than baseline scores *Mdn* = 9 resulting in a significance improvement of z = 3.029, *p* = 0.002, *r* = 0.24.

#### Knowledge of risk factors for NCDs

Table 3 shows the correct scores obtained for the heart disease knowledge questionnaire for the three timelines. The average total score at baseline was the lowest compared to 12 weeks and 24 weeks scores, indicating that less than half of participants were able to correctly answer the questions. Participants scored the lowest on dietary knowledge and risk factor knowledge about heart disease (three per cent on both). In contrast, 12 weeks scores were the highest of all three timelines. All the participants were unable to correctly answer question 6, which evaluates participants’ knowledge about diet and the difficulty mean (level of difficulty) is 0.66. More participants at 24 weeks compared to baseline were able to correctly answer the questions on heart disease. Interestingly, the same number of participants at 24 weeks and baseline correctly answered question 7. Question 7 is a heart disease risk factor question with a difficultly mean of 0.34.

**Table 3:** Coronary heart disease knowledge scores of all participants for each question

**Question**	**Baseline(%) *n* = 76**	**12 weeks(%) *n* = 40**	**24 weeks(%) *n* = 32**
1.Polyunsaturated fats are healthier for the heart than saturated fats	14 (18)	30 (75)	5 (16)
2.Women are less likely to get heart disease after menopause than before	15 (20)	11 (28)	6 (19)
3.Having had chickenpox increases the risk of getting heart disease	29 (38)	12 (30)	18 (56)
4.Eating a lot of red meat increases heart disease risk	37 (49)	26 (65)	18 (56)
5.Most people can tell whether or not they have high blood pressure	30 (40)	11 (28)	18 (56)
6.Trans-fats are healthier for the heart than most other kinds of fats	2 (3)	0 (0)	19 (59)
7.The most important cause of heart attacks is stress	2 (3)	3 (8)	1 (3)
8.Walking and gardening are considered types of exercise that can lower heart disease risk	67 (88)	37 (93)	23 (72)
9.Most of the cholesterol in an egg is in the white part of the egg	17 (22)	17 (43)	22 (69)
10.Smokers are more likely to die of lung cancer than heart disease	6 (8)	3 (8)	1 (3)
11.Taking an aspirin each day decreases the risk of getting heart disease	31 (41)	16 (40)	23 (72)
12.Dietary fibre lowers blood cholesterol	51 (67)	38 (95)	15 (47)
13.Heart disease is the leading cause of death in the United States	44 (58)	32 (80)	15 (47)
14.The healthiest exercise for the heart involves rapid breathing for a sustained period	33 (43)	17 (43)	12 (38)
15.Turning pale or grey is a symptom of having a heart attack	14 (18)	19 (48)	15 (47)
16.A healthy person's pulse should return to normal within 15 min after exercise	43 (57)	20 (50)	9 (28)
17.Sudden trouble seeing in one eye is a common symptom of having a heart attack	36 (47)	14 (35)	15 (47)
18.Cardiopulmonary resuscitation (CPR) helps to clear clogged blood vessels	4 (5)	2 (5)	11 (34)
19.HDL refers to “good” cholesterol, and LDL refers to “bad” cholesterol	14 (18)	5 (13)	10 (31)
20.Arterial defibrillation is a procedure where hardened arteries are opened to increase blood flow	5 (7)	4 (10)	12 (38)
21.Feeling weak, lightheaded, or faint is a common symptom of having a heart attack	33 (43)	24 (60)	14 (44)
22.Taller people are more at risk of getting heart disease	48 (62)	22 (55)	22 (69)
23.“High” blood pressure is defined as 110/80 (systolic/diastolic) or higher	30 (39)	22 (55)	22 (69)
24.Most women are more likely to die from breast cancer than heart disease	6 (8)	4 (10)	6 (19)
25.Margarine with liquid safflower oil is healthier than margarine with hydrogenated soy oil	26 (34)	28 (70)	8 (25)
26.People who have diabetes are at higher risk of getting heart disease	43 (57)	34 (85)	19 (59)
27.Men and women experience many of the same symptoms of a heart attack	32 (42)	22(55)	16 (50)
28.Eating a high fibre diet increases the risk of getting heart disease	31 (41)	36 (90)	21 (66)
29.Heart disease is better defined as a short-term illness than a chronic, long-term illness	37 (49)	28 (70)	17 (53)
30.Many vegetables are high in cholesterol	59 (78)	38 (95)	25 (78)
**Total scores**	**37**	**49**	**47**

#### Knowledge of physical activity

The participants' findings indicated very good knowledge about PA (Table 4). At baseline almost all the participants knew that PA improves health and general wellbeing. There were improvements in PA knowledge at 12 weeks compared to baseline. All the participants knew that PA contributes to cholesterol control. PA knowledge scores at 24 weeks were higher than baseline scores but lower than 12 weeks scores. Fewer people at 24 weeks compared to baseline knew that PA is not only good for some individuals. However, more participants at 24 weeks knew the frequency and intensity of PA engagement required to obtain health benefits.

**Table 4:** Physical activity knowledge scores of all participants for each question

	**Questions**	**Baseline(%) *n* = 76**	**12 weeks(%)*n* = 40**	**24 weeks(%)*n* = 76**
1	Physical activity is only good for some individuals: eg. elite sportspeople/ young people/ Caucasians	55 (72)	31 (78)	22 (67)
2	Exercise reduces high blood glucose (sugar) levels /diabetic complications	67 (88)	38 (95)	30 (91)
3	Physical activity of moderate intensity at least five times a week has positive effects on health	68 (90)	37 (93)	32 (97)
4	Exercise decreases physical dependence	54 (71)	38 (95)	25 (76)
5	Thirty minutes of physical activity everyday supports weight loss	72 (95)	35 (88)	28 (85)
6	Physical activity is good for your blood pressure no matter your age, weight, race, or gender	70 (92)	39 (91)	30 (91)
7	Physical activity causes/worsens pain	61 (80)	38 (95)	27 (82)
8	Exercise contributes to cholesterol control	72 (95)	40 (100)	30 (91)
9	Physical activity contributes to a better state of mind	74 (97)	39 (98)	32 (97)
10	Physical activity improves health and general wellbeing	75 (99)	39 (98)	32 (97)
**Total score**		**86**	**94**	**89**

### Integration of qualitative and quantitative results

Salient points from qualitative findings and quantitative results are integrated and presented in Table 5 using the HBM as a theoretical lens.

**Table 5:** Comparison of salient points from qualitative and quantitative results

Health belief model constructs	Integrated qualitative results and quantitative results
**Baseline results**	
*** Individual perceptions***	Secondary analysis of qualitative data and quantitative data reveal that perceived knowledge concurs with actual knowledge, as participants **perceived increased risk to NCDs** because of stress associated with poor-economic factors. Participants know that stress is the most important cause of heart disease
*** Modifying factors***	Quantitative determination reveals a high prevalence of NCD risk factors among participants. However, participants have limited **knowledge of NCD** risk factors. Participants reported knowing about NCDs after being diagnosed with an NCD condition. Participants scored high on **knowledge of PA;** on the contrary, they perceived a lack of **knowledge for PA**. Participants perceived encouragement from medical professionals
*** Likelihood of action***	Participants engage in low-intensity PA levels. **Perceived benefits** of PA engagement are socialising, stress management, and improved body function. **Perceived barriers** encapsulate discouragement by others in the community, low energy levels, and lack of knowledge about the type of activities to engage in
**12 weeks results**	
*** Individual perceptions***	Participants **perceived increased risk to NCDs** because of stress and modern lifestyle. They knew that stress is the most important cause of heart disease. Participants **perceived the seriousness of NCDs** as disease complications due to uncontrolled NCDs and NCD deaths. Most participants (80%) knew that heart disease is the leading cause of death in the USA
*** Modifying factors***	Results reveal a prevalence of NCD risk factors from biological measures. Participants perceived a lack of knowledge about the causes of NCDs. Survey findings congruently show low **knowledge of NCD** risk factors, participants particularly lacked knowledge about the difference between healthy fat and unhealthy fat. **Knowledge of PA** scored high; however, participants perceived a lack of knowledge about PA. Respondents report awareness from media to take up PA
*** Likelihood of action***	Objective measures show that participants mainly engaged in low-intensity PA levels. They **perceive the benefit** that PA engagement assists in stress reduction and that PA forms part of disease management. However, cultural **barriers to PA** engagement abound
**24 weeks results**	
*** Individual perceptions***	Participants **perceived increased risk to NCDs** as “tycoons’ sicknesses” because of lifestyle changes and stress. Most (97%) participants believe smokers are likely to die of lung cancer rather than heart disease. Also, 97% of participants believe stress is the most important cause of heart disease. **Perceived seriousness about NCDs** was ambiguous; some participants perceive that NCDs are serious conditions and can be fatal, while others perceive that NCDs are long-duration conditions and less likely to be fatal
*** Modifying factors***	Participants demonstrate poor **NCD knowledge**, they scored below average on heart disease knowledge. They perceive a lack of knowledge about the aetiology of NCDs, they knew family members diagnosed with an NCD condition. Participants scored high on the **PA knowledge** survey. Though results reveal confusion about the role of PA in disease management among participants
*** Likelihood of action***	Perceived benefits of PA include improved functional ability and stress management. Perceived barriers were lack of skill for PA and cultural barriers to PA

## Discussion

This study describes perceptions and knowledge of NCD risk factors and PA among women receiving standard clinic care in a low-resourced community setting, utilising the HBM as a lens for analysis of the data. The use of a convergent parallel mixed-method study enabled us to get a deeper understanding of the influence of standard clinic care on knowledge and perceptions of NCDs and PA of participants. Understanding the gap currently in the literature, will assist with the development of appropriate interventions. From the focus group discussions, participants perceived stress associated with living in a low-resourced environment as the most important contributing factor for an increased risk for NCDs. This perception is supported by the fact that most participants (97%) indicated that stress is an important cause of heart attacks in the heart disease-knowledge survey. These findings are supported by the literature. Findings from a review indicated that stress is moderately associated (Relative Risk (RR) = 4.7) with cardiovascular diseases among persons who already present with risk factors such as high blood pressure, high cholesterol levels, obesity, diabetes, and smoking compared to apparently healthy individuals (RR = 1.3) [25]. Since the mentioned risk factors were present in our population, it is understandable that stress is perceived as a contributing factor. Interventions addressing the risk factors may therefore also reduce stress within low-resourced populations presenting with risk factors for NCDs.

Follow-up findings at 12 and 24 weeks indicate that participants perceived unhealthy diets to be associated with modernisation and financial lack, as well as increased risk of NCDs. Similar findings were reported by respondents from a low-income peri-urban community. Their choice of diet was associated with their living conditions and poverty [26]. Knowledge survey results indicate that participants have limited dietary knowledge. Low-resourced communities consume high-energy, unhealthy diets because of affordability, accessibility and availability [27]. There seems to be a disconnect between perceptions and knowledge, which may be the driver of unhealthy diet patterns observed in low-resourced communities. Participants perceive that unhealthy diet and stress are the drivers for increased NCD risk. Although the standard clinic care program included workshops on healthy eating, the participants were not able to adjust their lifestyle to include a healthy diet. Similarly, Dlugonski and colleagues reported that single mothers considered unhealthy food choices as risk factors for NCDs and attributed lack of PA engagement to chronic stress [28]. Eckert and Kohler asserted that high NCD risk factors might be caused by rapid urbanisation, which fuels unhealthy behaviours such as reduced PA, smoking, alcohol abuse, and unhealthy diet [29]. Ongoing education programmes about the benefits of physical activity in the reduction of stress and risk factors for NCDs are necessary to raise awareness on how lifestyle behaviours can be improved. Current health promotion programs might need to be re-visited to provide more effective interventions to results in improved health outcomes.

Participants reported a lack of knowledge of risk factors for NCDs, having encountered NCDs for the first-time during diagnoses. This lack of awareness about NCDs is not uncommon in the South African context, as similar findings were reported by Sehole and Van Heever in their study [30]. Risk factors for NCDs were prevalent among this group of women, as observed in previous studies [31]. However, participants demonstrated good knowledge of PA for health but had limited knowledge about the type of PA they could engage in for health benefits. The type of PA activities they were familiar with are light activities that mainly encompassed the traditional female gender roles, including house chores mainly done in the morning, and active commuting. The implementation of regular moderate-to-vigorous PA could assist in reducing risk factors for NCDs and improve health [32]. Mass media campaigns among ethnic minority groups in the United States of America is a major driver for raising awareness about PA [33].

Participants perceived that being overweight increases NCD risk, similar to the findings of Nyberg and colleagues in a multi cohort population from Europe [34]. The participants from our study knew that NCDs are chronic conditions. Like the facility-based therapeutic intervention group, participants in this study had reductions in weight and waist circumferences at 24 weeks [35]. However, other NCD risk factors including DBP, SBP, BMI, and blood glucose remained unchanged – or worsened. Poor attendance of participants in NCD educational programmes [36] and low healthcare utilisation [37] have previously been reported to be partly responsible for poor health outcomes in low-resourced communities in South Africa. However, standard clinic care attendance monitoring was beyond the scope of this study. Prevalence of NCD risk factors, including low PA levels, was not influential in modifying health beliefs.

Participants perceived that PA is a form of socialising, which may assist in stress management. Stress management was particularly perceived as important. The results from the PA-knowledge survey indicate that most participants knew that PA improves mental state. While participants knew the benefit of PA engagement, they demonstrated lack of awareness about the type of PA they could safely engage in. The reason for these findings might be related to the lack of knowledge about regular physical activity that nurses receive during their training. Though participants did not perceive barriers to PA engagement, lack of PA-related skill may hinder regular PA engagement. The objective PA measurements show that, though participants met the WHO PA guidelines, they spent most of their time being sedentary and engaging in low-intensity activities. Similarly, Hill and colleagues found that, based on self-report methods, participants met PA levels; however, time spent sedentary was double that spent in moderate PA [38]. Group activities, walking [39], and house chores were the most preferred activities among participants [38]. Being among patients of similar chronic conditions motivates those patients, who prefer to join support groups as part of disease management [7, 28]. PA capacity building through practical programmes may improve regular PA engagement. The strength of the study is in the fact that this is the first study to investigate the perceptions and knowledge of risk factors for NCDs and PA by means of a mixed-method approach to obtain a deeper understanding about the perceptions within a low-resourced setting.

## Limitations

Compliance to the usual clinical care was not monitored, and objective PA measurements were not available for the 24 weeks post-baseline. The participants were not willing to wear the ActiHeart® devices again. This study is limited to one low-resourced municipality in the North West Province, as well as the use of a convenience sample does not allow for the data to be generalised to the rest of the country.

## Recommendations

Interventions and standard clinic care programme aimed to curb NCDs in low socio-economic communities should include aspects addressing behaviour-change strategies and perceptions of PA in accordance with culturally accepted physical activity. A focus on the reduction of stress and NCD risk-factor control should be included in  standard clinic care programmes. Mass media campaigns to promote PA behaviours in South Africa, with a focus on how to exercise, could raise PA awareness. The implementation of exercise specialists within the public health clinics, such as Biokineticists, might assist with changing perceptions about physical activity while also providing safe, individualised exercises to persons with risk factors for NCDs.

## Conclusion

In conclusion, the findings of this study indicate that the standard clinic care programme did not change the perceptions and knowledge of women from a low-resourced community regarding physical activity and risk factors for NCDs. Although the high level of physical activity knowledge should be sufficient to facilitate behaviour changes, after 24 weeks of the clinic care programme participants still reported confusion about risk factors for NCDs and the types of PA they can perform. Therefore, the usual standard clinic care alone does not lead to an understanding of risk factors for NCDs and positive perceptions of physical activity in this low-resourced community. Current clinic care programs should consider implementing supervised PA interventions by exercise experts to determine if perceptions about risk for NCDs and PA will be changed when persons from a low-resourced setting is exposed to experiential learning.

## Data Availability

The datasets analysed during the current study are not publicly available due to approval for sharing data publicly not being provided by the Health Research Ethics Committee but are available from the corresponding author on reasonable request.

## Abbreviations

AEE: Activity Energy Expenditure
B-Healthy: Be Healthy
BMI: Body Mass Index
CAQDAS: Computer-Assisted Qualitative Analyses Software
DBP: Diastolic Blood Pressure
FGDs: Focus Group Discussions
HBM: Health Belief Model
MVPA: Moderate To Vigorous Physical Activity
NCDs: Non-Communicable Diseases
NCT: Noticing Things; Collecting Things And Thinking About Things
NWU: North-West University
PA: Physical Activity
HBM: Health Belief Model
PAL: Physical Activity Level
PAR-Q: Physical Activity Readiness Questionnaire
W/H: Wait To Hip Ratio
SBP: Systolic
Total Chol: Total Blood Cholesterol
UK: United Kingdom
WC: Waist Circumference
W/H: Wait To Hip Ratio
